# The Acari Hypothesis, V: deciphering allergenicity

**DOI:** 10.3389/falgy.2024.1454292

**Published:** 2024-11-01

**Authors:** Andrew C. Retzinger, Gregory S. Retzinger

**Affiliations:** ^1^ Department of Emergency Medicine, Camden Clark Medical Center, West Virginia University, Parkersburg, WV, United States; ^2^ Department of Pathology, Feinberg School of Medicine, Northwestern University, Chicago, IL, United States

**Keywords:** the acari hypothesis, allergenicity, FReP, defensins, cystatins, peroxidases, chitinases, PR-10 proteins

## Abstract

The Acari Hypothesis posits that acarians, i.e., mites and ticks, are operative agents of allergy. It derived from observations that allergens are molecular elements of acarians or acarian foodstuffs. A corollary of The Hypothesis provides how acarian dietary elements are selected as allergens; namely, a pattern recognition receptor native to the acarian digestive tract complexes with dietary molecules problematic to the acarian. By virtue of its interspecies operability, the receptor then enables not only removal of the dietary elements by the acarian immune system, but also—should such a complex be inoculated into a human—production of an element-specific IgE. Because pattern recognition receptors bind to molecules problematic to the organism from which the receptors originate, it follows that molecules targeted by adaptive IgE, i.e., allergens, must be problematic to acarians. This claim is supported by evidence that host organisms, when infested by acarians, upregulate representative members of allergenic molecular families. Appreciation of the relationship between allergens and acarians provides insight well beyond allergy, shedding light also on the anti-acarian defenses of many living things, especially humans.

## 1 Introduction

In hope of identifying a unifying principle on the nature of allergenicity, existing allergy research has focused primarily on structural features of allergenic molecules. Indeed, most allergens derive from about 30–40 molecular families (1). Although within a given family significant sequential and structural homologies exist, allergenic molecules from different families vary greatly. Consequently, no truly unifying principle has come from structural analyses alone.

A teleological rationalization of allergenicity was proposed by Profet in 1991 (2). It postulates that allergens are molecules generally toxic to humans, with mast cell-mediated immune activation constituting a last line of defense against allergens as toxins. Indeed, such rationalization is supported by allergens attributable to venomous hymenopterans, e.g., *Apis mellifera*, the Western honeybee. With specific regard to *A. mellifera*, at least 12 allergens have been identified, all of which are expressed in the bee venom sac, venom duct and/or hypopharyngeal gland (3). Thus, at least in the case of Western honeybees, IgE does appear to target materials that might adversely affect human physiology.

Although the proposal of Profet accounts reasonably well for the allergenicity of molecules expressed by honeybees, it does not account for the inadequacy of the response directed against those molecules, i.e., the gross mechanical reflexes elicited by IgE do not effectively deter the sting of a bee. Furthermore, if honeybee venom is threatening to all humans, then why do so few humans elicit an IgE-mediated response following envenomation? In the context of otherwise benign allergens, the proposal is even more problematic. Pollens, as examples, cause debilitating IgE-mediated symptoms in many humans (4).

The Acari Hypothesis was conceived only very recently (5–8). Just as does the proposal of Profet, it foregoes structural considerations in the assignment of allergenicity using, instead, teleological considerations for that purpose. The Hypothesis proposes most, if not all, allergens derive from acarians or their foodstuffs. One of its corollaries offers mechanistic understanding relevant to the elicitation of IgE. It proposes an acarian pattern recognition receptor, perhaps a fibrinogen-related protein (FReP) operating within the acarian digestive tract, elicits IgE in humans following inoculation of the receptor and its associated materials (6).

**Figure 1:**
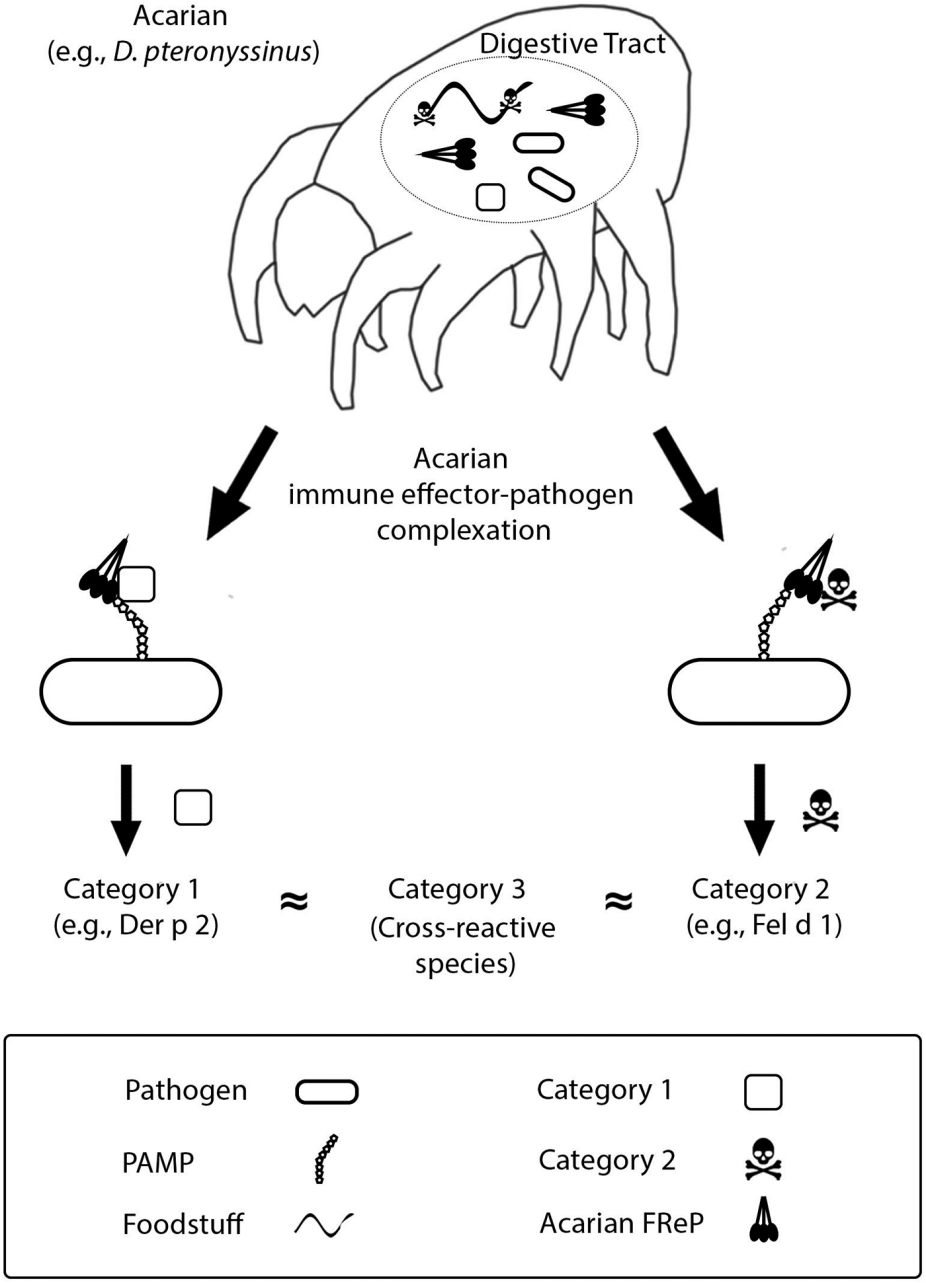
Deciphering allergenicity. See text and previous installments of this series for details (5–8).

Acarian FRePs, like many FRePs of other species, function as innate immune effectors (9). They bind and agglutinate materials that display molecular patterns dangerous to the acarian, earmarking those materials for phagocytosis and degradation (10). The fibrinogen-like domain of some FRePs contains multiple binding sites that can operate independently or synergistically (11). This enables them to recognize a vast array of endogenous and exogenous ligands. As envisaged per The Hypothesis, acarian FRePs may complex with 2 categories of allergens: (1) acarian immunoregulators, e.g., Der p 2, a major allergen of the human dust mite and a homolog of human myeloid differentiation factor 2 (12), and/or (2) molecular elements of acarian foodstuffs, Figure 1. Following inoculation into a human, complexes incorporating category 1 allergens prime the recipient for *felicitous* IgE directed against the acarian. In contrast, complexes incorporating category 2 allergens prime the recipient for *specious* IgE directed against the acarian dietary element. There is yet a third category of allergens: molecules that cross-react with IgE directed against category 1 or 2 allergens. Allergens from category 3 are always problematic. They often derive from invertebrates, e.g., insects, mollusks or helminths, because the allergenic molecules of mites, e.g., tropomyosin or paramyosin, share strong homology with the molecules of other invertebrates (13–17).

Indeed, the tropomyosin family exemplifies the complicated nature of IgE reactivity. Cockroach tropomyosins, which are homologous with acarian tropomyosins, are strongly allergenic (18–20). Thus, they may be category 3 allergens in persons sensitized to acarian tropomyosin. Cockroaches can also serve as phoretic hosts or foodstuffs for some acarian species (21, 22), rendering them a source of category 2 allergen. In short, depending upon the situation, cockroach materials are sometimes sensitizing and other times cross-reacting. This situational dependence undoubtedly also applies to other important allergens, even ones that derive from acarians.

In all instances, the human immune system construes the allergen as acarian in nature. Following subsequent exposure, the system activates reflexes intended to clear acarians from epithelial surfaces, e.g., cough, spit, itch, vomit, defecate, etc.

The Acari Hypothesis explains the observations that gave rise to the Profet proposal. It also accounts for the shortcomings of that proposal. Category 2 allergens are materials uniformly toxic to acarians, not to humans. Although some category 2 allergens are toxic to humans as well, e.g., melittin, most are problematic only to the acarian species from which the sensitizing complex originated.

Researchers studying plant immunity have classified 19 groups of molecules participating in plant defense against pathogens and phytophagous arthropods, i.e., pathogenesis-related proteins (PR 1–19) (23, 24). PR proteins are well-represented among human allergens (25), entirely consistent with the idea that IgE-targeted materials are toxic to acarians. From among the groups of PR proteins, the ones best exemplifying this unifying allergenic attribute are the defensins, cystatins, peroxidases, chitinases, and PR-10 proteins. That being the case, the remainder of this report focuses on them and three other families of common category 2 allergens: lipocalins, secretoglobins and palate lung nasal clone (PLUNC) proteins.

## 2 Defensins

The defensins are cysteine-rich, cationic immuno-polypeptides expressed by many plants and animals (26, 27). The group includes at least two protein superfamilies, each of which took a convergent evolutionary path predicated on the utility of a structural motif consisting of a cysteine-dense core and exteriorly displayed loops (28, 29). Defensins are well-represented within the human allergen database and include molecules from ragweed, peanut, celery and soybean (30).

Functionally, defensins have antimicrobial activity against a diverse microbial repertoire (31, 32). Their antimicrobial activity is believed to be due to direct interaction with cell membranes, causing lytic defects (33). Some defensins also inhibit α-amylase (34, 35), an enzyme commonly expressed within the digestive tract of arthropods, including acarians (36). Inhibition of α-amylase limits the ability of arthropods to digest foodstuffs. Consistent with an anti-acarian role, organisms upregulate defensins in response to acarian infestation. As an example, *A. mellifera* is subject to parasitism by the mite, *Varroa destructor* (37). Upon infestation by the mite, defensins are upregulated in *A. mellifera*, and increasing mite infestation increases defensin expression (38, 39). Because defensins inhibit acarian digestion, it is reasonable to assume acarian immune effectors operating within the acarian digestive tract complex with, and neutralize, defensins.

Plant defensins are categorized in the PR-12 grouping, and their role in defense against pathogens and phytophagous acarians is very well-established. As an example, *Arabidopsis* upregulates defensins in response to herbivory by the spider mite, *Tetranychus urticae* (40).

Humans also produce defensins, and there is evidence human defensins are involved in IgE-mediated disease. Human β-defensin-1 (HBD-1) and human β-defensin-2 (HBD-2) are innate immune effectors constitutively secreted by epithelial cells and leukocytes (41, 42). Consistent with the ideas that IgE-mediated disease is caused by acarian species and HBDs participate in human anti-acarian defense, levels of HBD-1 and -2 are altered in allergic disease (43, 44) and functional mutations in HBD-1 and -2 predispose children to both atopic dermatitis and asthma (45, 46).

## 3 Cystatins

Cystatins are a family of small proteins that are also well-represented in the database of the allergens of humans. They include molecules from plants, e.g., ragweed and kiwi (47–49), and mammals, e.g., canines and felines (50, 51). Plant cystatins are commonly referred to as phytocystatins and are assigned to the PR-6 grouping. The primary function of phytocystatins is inhibition of cysteine proteases, including those used by acarians to digest foodstuffs (52). Such inhibition deprives acarians of essential nutrients.

There are many examples of plant species that upregulate phytocystatins in direct response to acarian herbivory (53). As one example, phytocystatins expressed by barley protect against acarian parasitism (54). Indeed, transgenic expression of barley phytocystatins by wheat and maize confers anti-acarian protection (55). Maize, too, upregulates phytocystatins, especially in response to parasitism by mites *T. urticae* and *Oligonychus pratensis* (56). As another example, a cystatin expressed in the seeds of chestnuts inactivates Der f 1, the dominant allergen and well-described cysteine protease of the dust mite, *Dermatophagoides farinae* (57, 58).

*Dermatophagoides* is a genus of polyphagous synanthropic acarian proposed by The Acari Hypothesis to be a primary etiological agent of allergic disease. To digest human materials, the mites use cysteine proteases, like Der p 1 and Der f 1 (59). As do plants, humans express their own array of cystatins. Cystatin A is a cysteine protease inhibitor expressed by human keratinocytes (60). It neutralizes Der f 1 (61). Thus, just as phytocystatins defend plants against herbivory by phytophagous acarians, human cystatins defend humans against carnivory by anthropophagous acarians. Consistent with the proposal that mites are the etiological agents of allergy, a mutation of cystatin A is associated with development of atopic dermatitis (62). Another human cystatin, the salivary cystatin SN, has also been shown to inhibit mite proteases, and its expression is upregulated in asthmatic patients (63).

## 4 Peroxidases

Plant peroxidases are another family of molecules used by plants to defend against pathogens and phytophagous acarians. Peroxidases are assigned to the PR-9 grouping. They generate and detoxify peroxide, thereby regulating reactive oxygen species, which are directly toxic to numerous pathogens (23, 64, 65).

Many plant species use peroxidase in defense against acarian infestation. As examples, *Solanum dulcamara* upregulates peroxidase following its infestation by the gall mite, *Aceria cladophthirus* (66), and peroxidase activity confers resistance to *T. urticae* infestation by both the hops plant, *Humulus lupulus*, and the cassava plant, *Manihot esculenta* (67, 68). As in the cases of other PR proteins, peroxidases are well-represented among the allergens of humans. They include enzymes expressed by wheat, bananas and tomatoes (69–71).

Just as humans express analogs of plant defensins and phytocystatins, so, too, do humans express a peroxidase, and that peroxidase is involved in allergic inflammation. In humans, eosinophils mediate the late-stage inflammation characteristic of allergic disease (72). The cells drive inflammation via the release of chemical mediators, including eosinophilic peroxidase (73, 74). Obvious parallels between the secretory milieu of plants following acarian infestation and that of humans during bouts of allergic disease lend further credence to the proposal IgE is intended to target acarian species.

## 5 Chitinases

Chitin [β- (1–4)-poly-N-acetyl-D-glucosamine] is the primary structural component of acarian exoskeletons (75, 76). Because it is essential to acarian physiology, many organisms have evolved enzymes to degrade chitin, i.e., chitinases, to defend against acarian threats. As examples, maize and barley upregulate chitinase expression in response to infestation by phytophagous mites (56).

Using current nomenclature, and based on structural characteristics, chitinases are categorized into PR groupings 3, 4, 8 and 11 (77). Chitinases are well-represented in the database of known allergens of humans, and they include molecules from bananas, avocados, tomatoes and maize (78–81).

The human proteome includes two chitinases, chitotriosidase 1 (CHIT1) and acid mammalian chitinase (AMCase), and evidence indicates both enzymes play a role in IgE-mediated disease. CHIT1 is expressed by epithelial cells and monocytes (82). AMCase is expressed by monocytes/macrophages, lung epithelial cells and natural killer cells (83). Levels of CHIT1 and AMCase are elevated in persons with allergic disease (84–86). Additionally, genetic variations in AMCase predispose to bronchial asthma (87). These observations highlight yet other instances in which diverse organisms use a shared means to defend against acarian species.

## 6 PR-10 proteins and lipocalins

PR-10 proteins are another group of phytoproteins that participate in plant defense against pathogens and phytophagous acarians. Although their exact mechanism-of-action is unknown, it likely relates to a large hydrophobic cavity that spans the length of the molecules (88). The cavity, which binds a wide variety of ligands, is suspected to sequester and/or deliver hydrophobic molecules (88), perhaps ones vital to acarian metabolism/physiology.

The PR-10 proteins are well-represented in the database of known allergens of humans and include molecules from—among many other sources—birch pollen (Bet v 1), cherries, peanuts, tomatoes, celery and walnuts (89–93). They, too, are upregulated in response to acarian infestation. As an example, the subterranean clover, *Trifolium subterraneum*, upregulates a PR-10 protein in response to infestation by the red-legged earth mite, *Halotydeus destructor* (94).

Although PR-10 proteins do not have obvious mammalian analogues, they share structural homology with lipocalins (95), a family of proteins expressed by bacteria, plants and animals, including mammals (96, 97). Just as the structure of PR-10 proteins features a large central cavity for transporting and/or sequestering hydrophobic molecules, so does the structure of lipocalins (98). Given this feature, it is not unreasonable to think lipocalins share the anti-acarian activity of PR-10 proteins. Importantly, the secretory lipocalins of domesticated mammals are a major source of the allergens of humans (99). As examples, the lipocalins, Can f 1, Can f 2, Can f 4 and Can f 6 are allergens derived from canines (100).

Relatedly, humans secrete apolipoprotein D (apo D), a lipocalin, onto their epithelial surfaces (101, 102). Apo D, produced by eccrine glands, is the third most abundant protein in the sweat of healthy humans (102). In the context of The Hypothesis, it was recently theorized that the expansion of eccrine glands across the epidermal surface of catarrhine primates was a consequence of an acarian-related evolutionary pressure (8). As a consequence of that pressure, eccrine gland secretions evolved to become the primary anti-acarian defense of humans. Thus, the inclusion of apo D in those secretions is entirely consistent with an anti-acarian role for lipocalins.

Catarrhine primates are not the only mammals subject to acarian ectoparasitism. Just as catarrhines developed an expanded eccrine glandular system to deal with acarian threats, other mammals evolved other means to protect from acarians. And just as IgE informs on the anti-acarian activities of plant proteins, so, too, does it inform on the anti-acarian activities of mammalian proteins. Although the library of the allergens of humans continues to expand, mammalian allergens from two molecular families, secretoglobins and PLUNC proteins, are already well-described. These last two will be elaborated next, in the context of The Hypothesis.

## 7 Secretoglobins

Secretoglobins are another family of proteins against which humans produce IgE (103). They are expressed by mammals exclusively, and representative molecules are typically secreted onto epithelial surfaces (104). They are found in abundance within the surface liquids of the uterus, prostate, lungs, and lacrimal and salivary glands (104). Secretoglobins are released as disulfide-linked dimers that, like PR-10 proteins and lipocalins, have an internal hydrophobic cavity that binds and transports small hydrophobic molecules (104). Two secretoglobins are well-described allergens. Fel d 1, a major allergen from felines, is expressed in cat saliva. It is also secreted by cat sebaceous glands (105) and, therefore, present in exfoliated feline scale and related detritus. Ory c 3, a major allergen from rabbits, is present on rabbit fur (106). Although the functions of these allergenic molecules are not yet well-defined, their localization to epidermis ideally positions them to defend against acarian ectoparasitism.

The best studied human secretoglobin, secretoglobin family 1A member 1 (SCGB1A1), is expressed by clara cells, which are non-ciliated secretory cells of the upper airways (107). In comparison to healthy controls, asthmatics have lower levels of SCGB1A1 in both serum and bronchoalveolar fluid (108), and mutations in SCGB1A1 are associated with increased asthmatic risk (109). If, as proposed, asthma is an IgE-associated bronchospastic disease attributable to acarian species, and SCGB1A1 has an anti-acarian role, then one would expect both low levels and mutations of SCGB1A1 to influence the development of the disease.

## 8 PLUNC proteins

PLUNC proteins are a molecular family unique to air-breathing vertebrates (110). Like secretoglobins, they are secreted by mammals onto epithelial surfaces. PLUNC proteins are expressed abundantly in liquids that bathe oral, nasal and upper respiratory airways, where they act as surfactants and participate in mucosal immunity (111). Examples of major allergens include Fel d 8, which is secreted from the salivary glands of cats (112), and Ecu c 4, i.e., latherin, the dominant protein of equine sweat (113, 114).

Equine sweat is fundamentally different from human sweat, having developed via a distinct evolutionary pathway. Whereas human sweat is made by eccrine glands, equine sweat is made by apocrine glands and is much more protein-rich than its human counterpart (115). Like human sweat, equine sweat contains a lipocalin, in this case, the allergen, Ecu c 1 (114). Given the targeting of molecules in equine sweat by IgE, it is tempting to speculate that equine sweat, like human sweat, developed in response to acarian-related evolutionary pressure.

The most studied of the human PLUNC proteins is BPI fold-containing family A, member 1 (BPIFA1), a protein expressed in upper airways and involved in innate immunity (116, 117). Two experimental findings support the notion human PLUNC proteins participate in anti-acarian defense. Firstly, levels of BPIFA1 are markedly diminished in the airways of asthmatics (118). Secondly, BPIFA1 is cleaved and inactivated by Der p 1, an allergenic cysteine protease of the dust mite, *Dermatophagoides pteronyssinus* (119). Given the similarities of the epithelial secretions of diverse lineages of mammalian species and the proposed role of human epithelial secretions in anti-acarian defense, it appears the Acari significantly influenced mammalian evolution, particularly that having to do with epithelial surfaces.

## 9 Closing

By means of direct recognition and subsequent opsonization, some acarian immune effectors that operate in the acarian digestive tract neutralize pathogenic/toxic materials. According to The Acari Hypothesis, those effectors have interspecies operability and are involved in initiation of the adaptive IgE response of humans. If this is true, then it stands to reason non-acarian materials targeted by IgE must be pathogenic/toxic to acarians. Such reasoning is certainly valid for the allergens reviewed in this report: defensins, cystatins, peroxidases, chitinases, and PR-10 proteins.

As proposed and elaborated elsewhere, following a near extinction event suffered by our ancestral catarrhine primates, sweating evolved and displaced IgE as dominant anti-acarian defense (8). Because sweating has, in recent times, been abrogated by human hygiene, IgE has returned to the forefront as an operational anti-acarian defense. However, having evolved long ago to engage the parasitic predecessors of contemporary synanthropic mites, the IgE response is now largely anachronistic. As an example, *Dermatophagoides*, a genera of contemporary dust mites whose diet is exceptionally diverse and includes bacteria and fungi (120, 121), is a member of the family, *Pyroglyphidae* (122). *Pyroglyphidae* descended from Psoroptidia, a parvorder of acarians that parasitizes birds and mammals (123). *Pyroglyphidae* followed an exceedingly unusual evolutionary pathway, having evolved from parasites to free-living scavengers (124).

Because: (1) allergenicity depends upon the dietary choices of parasitizing acarians, and (2) the ancestors of synanthropic acarians that now cause allergy were carnivorous, our mammalian ancestors might have been at risk for IgE-mediated red meat allergy had they not themselves expressed galactose-α-1,3-galactose (α-gal) (125). It was only after catarrhine primates stopped expressing α-gal that the possibility of allergy to the disaccharide could exist. Then, only after parasitic mites became polyphagous did allergies beyond red meat become possible. In short, allergies suffered by humans today are consequences of modern-day hygiene and modern-day diversification of the diets of domestic *Pyroglyphidae*.

The Acari Hypothesis is yet unproven. It assumes a role for FRePs in the development of mammalian Th2 immunity. Further, it attributes to mammals the ability to utilize acarian FRePs to direct the IgE arm of the Th2 response. Extensive testing is required to determine whether either of these is valid. If both are valid, however, then elucidation of how IgE is elicited yields information relevant to more than just allergy. Perhaps most importantly, it facilitates the identification of all manner of molecules and mechanisms used by lifeforms in defense against acarians. Although this report focuses on endogenous defensive molecules of plants and animals, the defenses of plants and animals likely also include materials expressed by constituents of their respective microbiomes. As demonstrated by the pathophysiology of atopic dermatitis, IgE targets an extensive array of molecules expressed by *Malassezia* spp., indicating this genus must be problematic to acarians (126, 127).

Amongst fungal species, *Malassezia* is unusual in that members of the genus lack a gene for fatty acid synthase (128). Absent this enzyme, *Malassezia* cannot generate fatty acids essential for survival. Consequently, the fungus has an absolute requirement for exogenous fatty acids. Human sebum is composed primarily of triglycerides and free fatty acids (129). Given the nutrient requirements of *Malassezia,* it comes as no surprise secretion of sebum onto mammalian epidermis yields an environment especially conducive to malassezial growth. Indeed, *Malassezia* is the dominant eukaryote of the epidermal microbiome of adult humans (130).

The apparent anti-acarian activity of *Malassezia* as well as the eminent suitability of mammalian skin for malassezial growth makes it likely the relationship between mammals and *Malassezia* is one of mutualism, wherein mammals supply *Malassezia* with essential lipids and the fungus defends humans from acarians. A role for sebaceous glands in human anti-acarian defense is supported by the epidemiology of allergy, the incidence of which is highest during childhood (131–133) and lowest following puberty, when sebaceous gland output and *Malassezia* colonization rise precipitously (134–137). The impact of the mutualism of humans and *Malassezia* on the epidemiology of allergy is elaborated in the next installment of this series (ACR, submitted).

## Data Availability

The original contributions presented in the study are included in the article/Supplementary Material, further inquiries can be directed to the corresponding author.
